# Differences in Quality of Life Between Patients on Peritoneal Dialysis and Hemodialysis in Saudi Arabia: A Cross-Sectional Study

**DOI:** 10.7759/cureus.78328

**Published:** 2025-02-01

**Authors:** Hassan A Alhabib, Ali Alhabib, Nawaf S Mohamed, Lina F Serhan, Abdullah Alabdullah, Sara Alramadhan, Ahmed j Al Habeeb

**Affiliations:** 1 Nephrology, Jubail General Hospital, Jubail, SAU; 2 College of Medicine, Almaarefa University, Riyadh, SAU; 3 Medicine, Almaarefa University, Riyadh, SAU

**Keywords:** hemodialysis, nausea, nephrology, peritoneal dialysis, saudi arabia

## Abstract

Background: The World Health Organization defines health as complete physical, mental, and social well-being. Chronic diseases impact health-related quality of life (QoL). Health services for ill populations, including end-stage renal disease (ESRD), focus on QoL issues and mental health promotion. Kidney transplantation is the optimal form, but most patients require dialysis, which can be hemodialysis (HD) or peritoneal dialysis (PD). It is believed that choosing the proper modality will help in improving patient’s QoL and satisfaction rate. To help patients select the mode of dialysis that best meets their needs while they have ESRD, this study intends to investigate differences in QoL between dialysis modalities.

Methodology: It was a multicenter cross-sectional study conducted in Saudi Arabia, focusing on HD and PD patients in specific hospitals. Data was collected through a specially designed online questionnaire and analyzed with IBM SPSS 29 (IBM Corp. Released 2023. IBM SPSS Statistics for Windows, Version 29.0.2.0 Armonk, NY: IBM Corp).

Results: Our study, including 307 dialysis patients in Saudi Arabia, revealed a male predominance (56.0%), primarily aged 45-64 years (43.0%), with 91.5% Saudi nationals. Employment varied (27.4% professionals), and most earned 10,000-20,000 SAR monthly (50.5%). Positive life changes post-dialysis were reported by 98.7%, with 94.5% not requiring post-dialysis absence from work. Incenter HD patients constitute 45.3% while PD patients constitute 54.7% of the study sample. Physical well-being showed significant differences in nausea (p=0.035). Social and emotional well-being exhibited acceptance variations (p=0.012) and coping satisfaction (p=0.014). Linear regression indicated monthly income impact on HD QoL (B=-3.706, p=0.075), while age influenced PD QoL (B=0.113, p=0.927).

Conclusion: Our study underscores positive life changes and minimal work absence is prevalent among dialysis patients. PD exhibits higher nausea and lower family acceptance. HD participants report greater coping satisfaction. The study reveals nuanced differences in well-being dimensions, contributing insights for tailored interventions and patient-centered care in renal replacement therapies.

## Introduction

Health is defined by the World Health Organization (WHO) as a state of complete physical, mental, and social well-being, not merely the absence of disease or infirmity. Since chronic diseases impact health-related quality of life (HRQOL), HRQOL has become a key outcome measure in disease management [1]. Globally, an estimated five to 10 million people die every year from chronic kidney disease (CKD), which is an irreversible illness that progressively damages the patients’ health and quality of life (QoL) [2]. In this context, health services for ill populations, including those with end-stage renal disease (ESRD), have drawn attention to quality-of-life issues and the promotion of mental health [3]. Kidney transplantation is the optimal form of renal replacement therapy, offering patients with ESRD the best prognosis for survival and quality of life (QoL). Despite this, due to the rapid rise in the incidence of ESRD and the long waiting list for transplantation, most patients with ESRD will require some form of dialysis during their lifetime [4]. The latter can be further subdivided into hemodialysis (HD) and peritoneal dialysis (PD). Regarding HD, it is typically performed three times a week using a dialysis machine in an outpatient facility under the supervision of nurses, while PD patients receive training from professional nursing staff and typically self-administer dialysis at home, either autonomously or with the help of a caregiver [5]. Approximately 10-20% of patients on PD are transferred annually to HD due to technique failure, while a much smaller proportion of patients switch from HD to PD, predominantly due to vascular access issues, cardiac disease, or patient preference [6]. In the more recent Broadening Options for Long-Term Dialysis in the Elderly (BOLDE) Study, older patients on PD experienced less illness intrusion compared to those on HD, after adjusting for comorbidities and other confounders [7]. Several studies have shown decreased QoL and increased depression in the HD patient population, with poor QoL itself also linked to increased complications such as depression, malnutrition, and even higher mortality rates [8]. Furthermore, before selecting a method of renal replacement therapy, patients should be thoroughly informed of all available treatment options, without pressure to select or reject a particular method. The choice of treatment should be an independent, conscious, and optimal decision for the patient [9]. It is believed that choosing the appropriate modality will help improve the patient’s QoL and satisfaction rate. We hypothesize that patients on PD may have a better QoL than those on HD. Therefore, the main aim of this study is to determine the differences in QoL between patients undergoing HD and PD in Saudi Arabia.

## Materials and methods

It was a multicenter cross-sectional study conducted in Saudi Arabia targeting patients undergoing HD and PD at the following centers: Saad Muajil Kidney Center, Qatif Central Hospital, King Saud Medical City, and Albaha Hospital in 2023. The inclusion criteria for the study were ESRD patients undergoing HD and PD at Saad Muajil Kidney Center, Qatif Central Hospital, King Saud Medical City, and Albaha Hospital in 2023. The exclusion criteria for the study included patients with ESRD not undergoing HD or PD, patients residing outside Saudi Arabia, and patients with neurological ailments, with data collected from at least 300 patients of both genders using a non-probability quota sampling technique. The data were collected through an online-based questionnaire using Google Forms, available in both English and Arabic (Table 7 of Appendices) [10]. The questionnaire contained sociodemographic data along with four sections: the first section focused on the physical well-being of the patient, the second on social/family well-being, the third on emotional well-being, and the fourth on functional well-being. An additional fifth section was included to address concerns regarding the patient’s employment status, mood, and spiritual beliefs. All data were cleared, coded, and entered using the International Business Machines (IBM) Statistical Package for the Social Sciences (SPSS) Software, version 29.0.0 (IBM Corp. Released 2020. IBM SPSS Statistics for Windows, Version 27.0. Armonk, NY: IBM Corp). The results were presented in tables and graphs, showing frequencies and percentages, and appropriate statistical tests of significance were used to test for associations. First, descriptive analysis was conducted to summarize the demographic characteristics of the participants, including age, gender, and other features, providing an overview of the study population. Subsequently, inferential analyses, such as the Independent Sample T-Test (for two groups), were employed to examine differences between PD and HD. Linear logistic regression was used to identify predictors of QoL. Statistical significance was set at a p-value of 0.05 or lower, with a 95% confidence interval. All research writing adhered to the fundamental ethical standards and policies of the Institutional Review Board (IRB) at Almaarefa University (IRB23-057, dated 19/06/2023). All participants' information and data were collected after obtaining consent and maintaining strict privacy throughout the study period.

## Results

Our study included a total of 307 patients undergoing dialysis treatment in Saudi Arabia (Table 1). The gender distribution shows a slight male predominance (56.0%, n=172), with diverse age groups, most prominently 45-64 years old (43.0%, n=132). Saudi nationals constitute the majority (91.5%, n=281), with a predominant marital status of married individuals (70.4%, n=216). Residents from the central region are most represented (59.9%, n=184). Employment status varies, with 27.4% (n=84) being professional employees. Monthly income is predominantly between 10,000 and 20,000 SAR (50.5%, n=155). Notably, 98.7% (n=303) reported a positive life change after changing dialysis, and 94.5% (n=290) did not require time off from work or school within one month after dialysis.

**Table 1:** Sociodemographic and other parameters of dialysis patients assessed for quality of life (n=307)

Parameters		Frequency (n=307)	Percent
Gender	Female	135	44.0
	Male	172	56.0
Age	5-14 years‎	14	4.6
	15-24 years	26	8.5
	25-44 years	84	27.4
	45-64 years	132	43.0
	>65 years	51	16.6
Nationality	Non-Saudi	26	8.5
	Saudi	281	91.5
Marital status	Single	56	18.2
	Married	216	70.4
	Widow/divorced	35	11.4
Region	North/West/South	17	5.5
	East	106	34.5
	Central	184	59.9
Employment status	Unemployed	10	3.3
	Student	39	12.7
	Housewife	60	19.5
	Professional employee	84	27.4
	Private employee	55	17.9
	Retired	59	19.2
Monthly income	<5000 SAR	103	33.6
	10,000-20,000 SAR	155	50.5
	20,000-25,000 SAR	42	13.7
	>25,000 SAR	7	2.3
Smoking	No	211	68.7
	Yes	96	31.3
Did you have to be absent from your work, school, or college after dialysis within one month?	No	290	94.5
	Yes	17	5.5
Has your life changed for the better after you changed the type of dialysis?	No	4	1.3
	Yes	303	98.7

Table 2 shows various dialysis-related features among participants. The majority undergo PD (54.7%, n=168), compared to HD (45.3%, n=139). A substantial portion (74.9%, n=230) continues with the same dialysis type, primarily due to satisfaction with the treatment (25.4%, n=78) and its positive impact on lifestyle (20.5%, n=63). Participants who switched dialysis (24.8%, n=76) often did so for reasons such as unsatisfactory results (7.8%, n=24) or pain associated with the previous method (2.0%, n=6). Notably, 10.4% (n=32) had a previous kidney transplant. A significant portion of participants switched from HD to PD (38.8%, n=119). Various dialysis centers were utilized, with King Saud Medical City being the most common (45.0%, n=138). The duration of dialysis varied, with a significant portion having undergone treatment for over a year (52.7%, n=162).

**Table 2:** Different dialysis-related features among participants (n=307)

Dialysis-related features		Frequency (n=307)	Percent
Dialysis type	Hemodialysis	139	45.3
	Peritoneal dialysis	168	54.7
Is it the same as the current dialysis?	No	76	24.8
	Yes	230	74.9
If yes, why did you choose the same dialysis?	Best dialysis treatment	32	10.4
	Dialysis is giving good results	22	7.2
	Helping toward a better lifestyle	63	20.5
	Satisfied with the treatment	78	25.4
If not, why choose the other type?	Dialysis was not giving results	24	7.8
	Not satisfied with the current dialysis treatment	28	9.1
	Old dialysis was painful	6	2.0
Have you changed your type of dialysis?	From hemodialysis to peritoneal dialysis	119	38.8
	From peritoneal dialysis to hemodialysis	51	16.6
	Not changed	137	44.6
Kidney transplant before	No	275	89.6
	Yes	32	10.4
Center for dialysis	Dammam Central Hospital (Kanoo Kidney Center)	42	13.7
	Jubail Hospital Saad Muajil Kidney Center	47	15.3
	King Khaled Hospital in Riyadh	76	24.8
	King Saud Medical City	138	45.0
	Others	4	1.3
How long you are taking dialysis treatment	<3 months	30	9.8
	3-6 months	39	12.7
	6-12 months	76	24.8
	1-3 years	56	18.2
	3-5 years	42	13.7
	5-10 years	34	11.1
	>10 years	30	9.8

**Figure 1:**
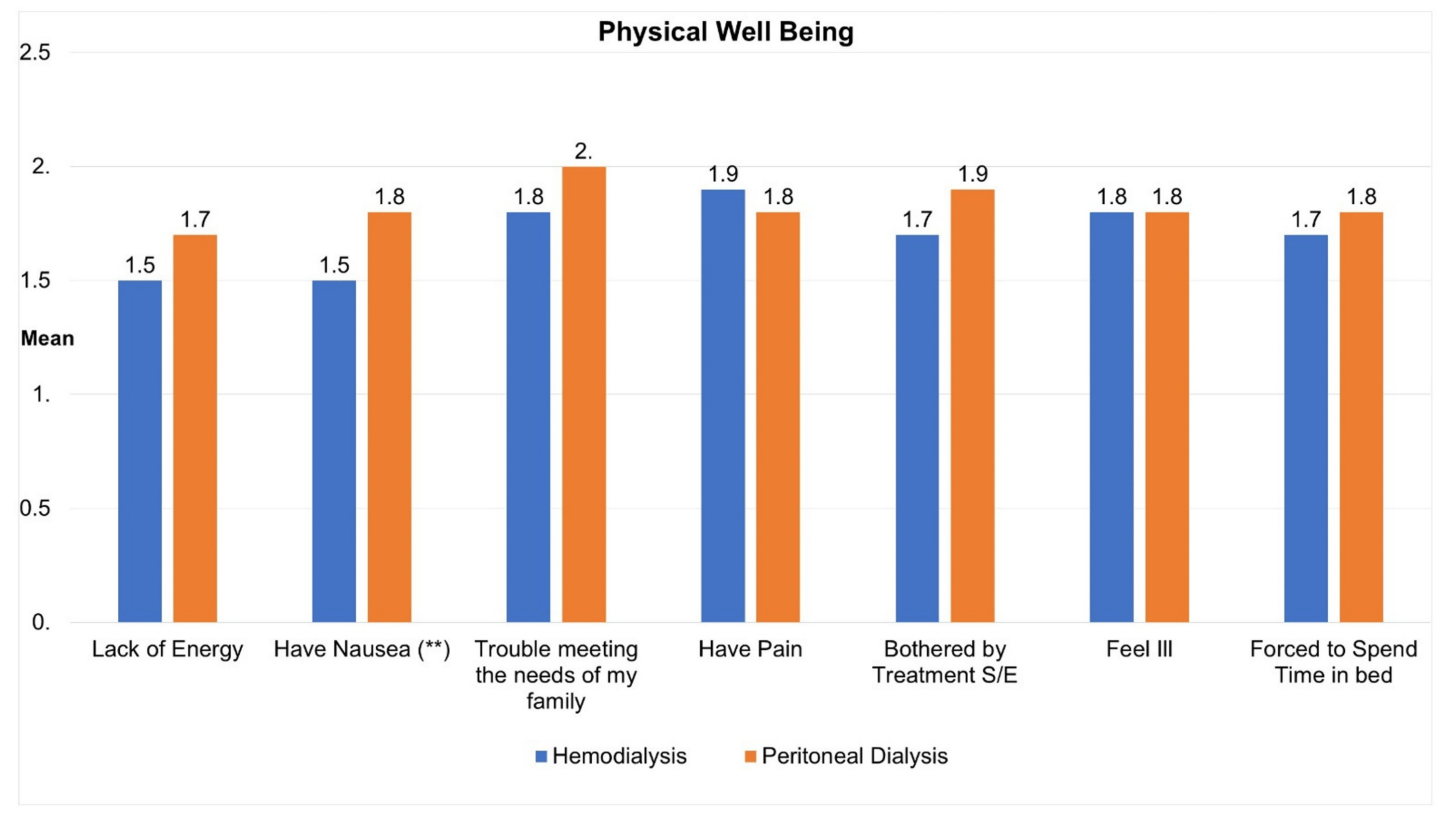
Mean physical well-being of participants according to dialysis type (independent samples T-test) **The incidence of nausea was notably higher in patients receiving peritoneal dialysis in comparison to those undergoing hemodialysis (p<0.05).

Figure 1 shows the physical well-being of participants based on dialysis type, comparing HD (N=139) and PD (N=168). Participants on HD reported a mean lack of energy score of 1.54 (SD=1.3), slightly lower than those on PD, with a score of 1.77 (SD=1.3) (p=0.146). Nausea was more pronounced in PD (mean=1.80, SD=1.3) compared to HD (mean=1.50, SD=1.2), showing a significant difference (p<0.05). Other parameters, such as family-related challenges, pain, side effects, feeling ill, and time spent in bed, did not exhibit statistically significant differences between the two groups.

**Figure 2:**
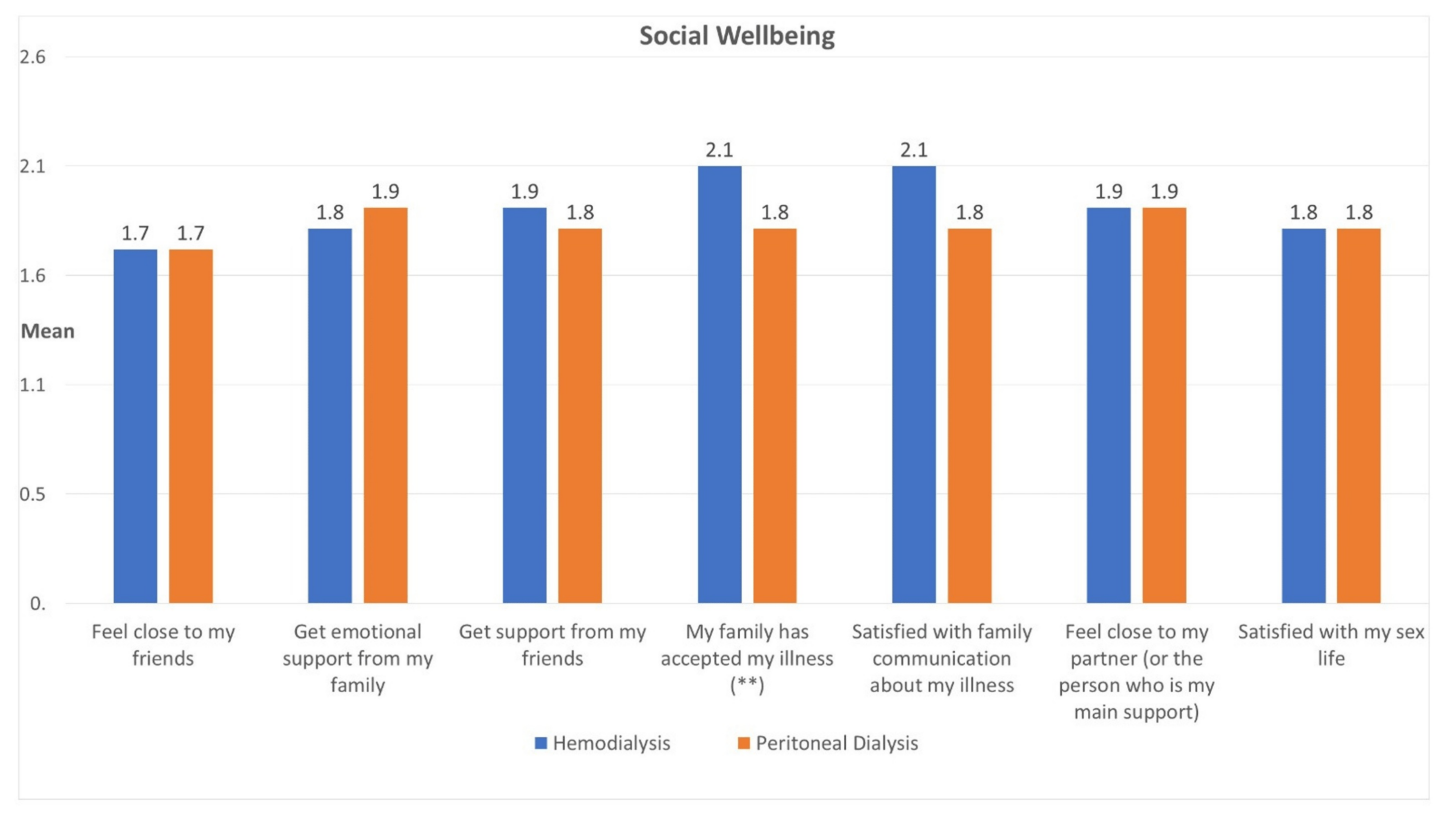
Mean social well-being of participants according to dialysis type (independent sample T-test) **The level of family acceptance regarding the illness was significantly elevated among hemodialysis patients relative to those on peritoneal dialysis (p<0.05).

Figure 2 shows the social well-being of participants based on two dialysis types. Participants on HD and PD reported similar levels of closeness to friends, emotional support from family, and support from friends. However, there were notable differences in certain aspects. HD participants reported significantly higher acceptance of their illness by family (mean=2.13, SD=1.2) compared to PD participants (mean=1.78, SD=1.1) (p<0.05). Family communication satisfaction regarding the illness showed a trend toward near significance (p>0.05), with HD patients being more satisfied (mean=2.1, SD=1.2).

**Figure 3:**
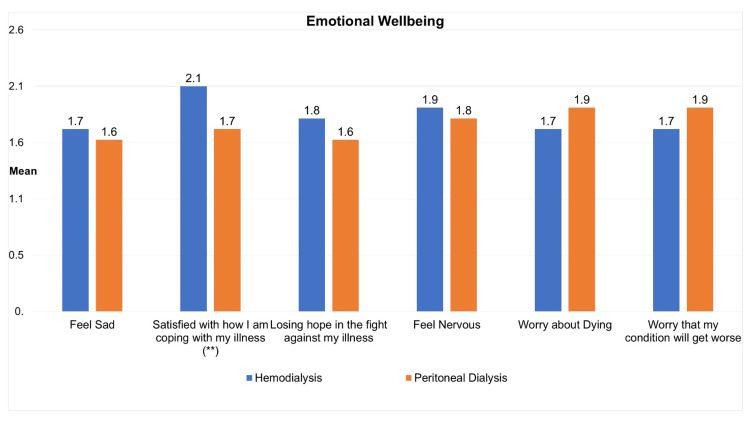
Mean social well-being of participants according to dialysis type (independent samples T-test) **Participants on hemodialysis reported a significantly greater level of coping satisfaction than their counterparts on peritoneal dialysis (p<0.05).

Figure 3 shows the emotional well-being of participants according to dialysis type. Participants in HD and PD reported almost similar levels of sadness, nervousness, worrying about dying, and worrying about their condition getting worse. However, significant differences emerge in coping with illness, where HD participants (mean=2.09, SD=1.2) expressed higher satisfaction compared to PD (Mean=1.73, SD=1.2) (p<0.05). Losing hope in the fight against illness did not show a statistically significant difference. The overall emotional well-being score also did not exhibit a significant difference between HD (mean=11.20, SD=4.6) and PD (mean=10.70, SD=5.1) (p>0.05).

Table 3 shows the functional well-being of participants based on dialysis type. Participants on HD and PD demonstrated nearly identical levels of ability to work (including work at home), fulfillment in their work, acceptance of illness, sleeping well, and enjoying usual activities. Differences in enjoying life and contentment with their current QoL did not reach statistical significance. The overall functional well-being score also did not show a significant difference between HD (mean=11.35, SD=5.1) and PD (mean=11.08, SD=5.1) (p=0.641).

**Table 3:** Functional well-being of participants according to dialysis type ^a^The alpha significance value (p-value) related to the mean comparisons of functional well-being parameters between the hemodialysis and peritoneal dialysis groups. A p-value of less than 0.05 is regarded as statistically significant.

Physical well-being of participants according to dialysis type	Hemodialysis (N=139)	Peritoneal dialysis (N= 168)	^a^Sig. value
	Mean (SD)	Mean (SD)	
I am able to work (include work at home)	1.65 (1.2)	1.87 (1.2)	0.136
My work (include work at home) is fulfilling	1.81 (1.2)	1.85 (1.1)	0.780
I am able to enjoy life	1.96 (1.3)	1.85 (1.1)	0.426
I have accepted my illness	1.91 (1.3)	1.94 (1.2)	0.817
I am sleeping well	1.78 (1.2)	1.92 (1.2)	0.326
I am enjoying the things I usually do for fun	1.90 (1.2)	1.77 (1.2)	0.372
I am content with the quality of my life right now	1.99 (1.2)	1.74 (1.2)	0.082
Overall functional well-being	11.35 (5.1)	11.08 (5.1)	0.641

Table 4 shows additional concerns related to well-being and quality of life among participants based on two dialysis types. Participants on HD and PD reported nearly identical levels of feeling peaceful, having a reason for living, leading a productive life, and finding meaning and purpose. No statistically significant differences were observed in the sense of harmony, comfort in faith, or belief in the positive outcome of illness. The overall score for additional concerns showed no significant difference between HD (mean=22.88, SD=8.3) and PD (mean=22.22, SD=9.4) (p=0.521).

**Table 4:** Additional concerns for well-being and better quality of life of participants according to dialysis type ^a^The alpha significance value (p-value) related to the mean comparisons of functional well-being parameters between the hemodialysis and peritoneal dialysis groups. A p-value of less than 0.05 is regarded as statistically significant.

Social well-being of participants according to dialysis type	Hemodialysis (N=139)	Peritoneal dialysis (N= 168)	^a^ Sig. value
	Mean (SD)	Mean (SD)	
I feel peaceful	1.99 (1.3)	1.75 (1.3)	0.120
I have a reason for living	2.02 (1.2)	1.83 (1.3)	0.208
My life has been productive	1.88 (1.1)	1.83 (1.2)	0.711
I have trouble feeling peace of mind	1.78 (1.3)	1.70 (1.2)	0.573
I feel a sense of purpose in my life	1.92 (1.2)	1.82 (1.2)	0.496
I am able to reach down deep into myself for comfort	1.91 (1.1)	1.88 (1.3)	0.821
I feel a sense of harmony within myself	1.91 (1.2)	1.90 (1.2)	0.915
My life lacks meaning and purpose	1.73 (1.2)	1.75 (1.2)	0.871
I find comfort in my faith or spiritual beliefs	1.99 (1.3)	1.96 (1.3)	0.820
I find strength in my faith or spiritual beliefs	1.96 (1.1)	2.04 (1.3)	0.591
My illness has strengthened my faith or spiritual beliefs	1.91 (1.2)	1.90 (1.2)	0.957
I know that whatever happens with my illness, things will be okay	1.86 (1.4)	1.86 (1.4)	0.970
Overall score for additional concerns	22.88 (8.3)	22.22 (9.4)	0.521

Table 5 shows the association between different employment statuses and the type of dialysis among participants, comparing HD (N=139) and PD (N=168). The distribution of unemployed participants is 70.0% for HD and 30.0% for PD. Students make up 38.5% of HD and 61.5% of PD; housewives account for 40.0% of HD and 60.0% of PD; professional employees are equally distributed at 50.0% of both HD and PD; private employees comprise 45.5% of HD and 54.5% of PD; and retired individuals represent 44.1% of HD and 55.9% of PD. The associations reveal no statistically significant difference in employment status between HD and PD groups (p=0.473, Fisher’s Exact Test).

**Table 5:** Association between different employment status and type of dialysis

Different employment status		Type of dialysis		Total
		Hemodialysis (N=139)	Peritoneal dialysis (N=168)	
Unemployed	N	7	3	10
	%	70.0%	30.0%	100.0%
Student	N	15	24	39
	%	38.5%	61.5%	100.0%
Housewife	N	24	36	60
	%	40.0%	60.0%	100.0%
Professional employee	N	42	42	84
	%	50.0%	50.0%	100.0%
Private employee	N	25	30	55
	%	45.5%	54.5%	100.0%
Retired	N	26	33	59
	%	44.1%	55.9%	100.0%

Table 6 shows the predictors for better QoL among HD and PD patients using a linear regression model. For HD, important predictors include high monthly income (B=-3.706, p=0.075) and being a smoker (B=3.032, p=0.301). A one-unit increase in the duration of dialysis is associated with a 0.601-unit improvement in QoL (p=0.397). For PD, being male (B=5.649, p=0.056) and not being a smoker (B=-4.818, p=0.151) show some trends. A one-unit increase in age is associated with a 0.113 increase in QoL (p=0.927). Overall, the models for both HD and PD are statistically significant (p<0.001), with the predictors collectively explaining variations in QoL among patients.

**Table 6:** Predictors of better quality of life among both types of dialysis patients (linear regression model)

Functional well-being of participants according to dialysis type	Unstandardized coefficients		Standardized coefficients	Sig.	95.0% CI	
	B	SE	Betas		Lower bound	Upper bound
Predictors for hemodialysis						
Constant	70.275	5.582		0.000	59.232	81.318
Gender (Male)	-1.833	3.126	-0.066	0.559	-8.018	4.353
Age	1.057	1.298	0.076	0.417	-1.510	3.625
Nationality (Saudi)	0.750	3.992	0.017	0.851	-7.147	8.648
Marital status (married)	1.346	1.798	0.071	0.455	-2.210	4.903
High monthly income	-3.706	2.063	-0.194	0.075	-7.788	0.376
Smoker (yes)	3.032	2.923	0.113	0.301	-2.750	8.814
Duration of dialysis	0.601	0.708	0.077	0.397	-.799	2.002
Kidney transplant (Yes)	-1.472	4.280	-0.030	0.731	-9.939	6.995
Predictors for peritoneal dialysis						
Constant	62.842	5.958		0.000	51.074	74.609
Gender (male)	5.649	2.939	0.189	0.056	-0.156	11.453
Age	0.113	1.233	0.008	0.927	-2.323	2.548
Nationality (Saudi)	4.330	4.716	0.075	0.360	-4.985	13.644
Marital status (married)	0.653	1.872	0.030	0.728	-3.043	4.350
High monthly income	0.811	1.727	0.042	0.639	-2.600	4.222
Smoker (yes)	-4.818	3.339	-0.135	0.151	-11.412	1.777
Duration of dialysis	-0.286	0.709	-0.034	0.687	-1.686	1.115
Kidney transplant (Yys)	-1.358	3.574	-0.030	0.705	-8.417	5.701

## Discussion

ESRD is characterized by kidney abnormalities, either structural or functional, and a reduced glomerular filtration rate (GFR), or both, lasting longer than three months. Similarly, Singh et al. (2008) stated that CKD is defined as a GFR below 60 mL/min/1.73 m² for three or more months, with or without kidney damage [11]. Bello et al. (2022) reported that several studies from nine countries indicated a mean prevalence of 55% for dialysis among patients with ESRD [12]. Various factors influencing QoL include demographic variables, duration of dialysis, and comorbidities [13]. Thus, our study comprehensively analyzes the QoL in Saudi Arabian dialysis patients, examining demographics, dialysis-related factors, and well-being dimensions. It also explores associations with employment status, dialysis type, and QoL predictors.

Notably, the demographic profile of the participants reveals a slight male predominance, consistent with global trends in dialysis, where men make up a larger proportion of the population. Lewandowski et al. (2023) showed that, globally, men predominantly undergo kidney replacement treatments, including dialysis and transplantation, with a 60:40 ratio compared to women [14]. The diverse age distribution, with a prominence of individuals aged 45-64, reflects the broad spectrum of age groups affected by kidney disease and the rapid loss of renal function in older age. Similarly, Toyama et al. (2020) found that older age is associated with faster loss of kidney function [15]. The majority being Saudi nationals, predominantly married, and residing in the central region underscores the local representation of the study population. The prevalence of professional employees and a monthly income between 10,000 and 20,000 SAR provides context for the participants' socioeconomic status. Remarkably, a high percentage reported a positive life change after changing dialysis, highlighting the potential impact of treatment on overall well-being.

Notably, the majority opting to continue with the same dialysis type highlights satisfaction with treatment and its positive impact on lifestyle. Reasons for switching, such as unsatisfactory results or pain associated with the previous method, provide insight into patient preferences and the importance of tailoring treatment to individual needs. A previous study by Jaar et al. (2009) showed that infectious peritonitis was the leading cause of switching from PD to HD [16]. The prevalence of previous kidney transplants adds complexity to the patient population, indicating a history of varied renal interventions. The choice of different dialysis centers, with King Saud Medical City being the most common, suggests accessibility and regional preferences. The duration of dialysis exceeding a year for a significant portion of participants emphasizes the chronic nature of their condition.

Regarding physical well-being, the mean lack of energy scores and overall physical well-being scores show no significant differences, with nausea being more pronounced in PD. This aligns with a study by Dial et al., in which patients with PD were reported to experience more gastrointestinal (GI) symptoms (including nausea and vomiting) compared to those on HD [17]. The lack of significant differences in family-related challenges, pain, side effects, feeling ill, and time spent in bed suggests a comparable impact on various aspects of physical well-being.

Moreover, social well-being shows similar levels of closeness to friends, emotional support from family, and support from friends between the two dialysis groups. However, notable differences emerge in family acceptance of illness, with HD participants reporting significantly higher levels. The trend toward significance in family communication satisfaction suggests potential variations in the support systems and communication dynamics within families of patients on different dialysis modalities. Similarly, Theodoritsi et al. (2016) highlight the role of family in supporting HD patients, noting that social support from family, friends, coworkers, spiritual guides, health professionals, and community members plays a significant role in influencing well-being [18].

Moreover, emotional well-being reveals comparable levels of sadness, nervousness, and worry about the future between the HD and PD groups. However, HD participants report higher satisfaction with coping with their illness. This finding aligns with previous research emphasizing the psychological impact of coping mechanisms and emotional resilience in patients undergoing dialysis. In contrast, Aljenaidi et al. (2023) found that patients starting PD had better QoL scores across all domains (including higher satisfaction with coping with illness) compared to patients starting HD [19]. However, Zazzeroni et al. (2017) showed that patients with PD had significantly higher levels of depressive symptoms than those with HD [20].

Moreover, functional well-being shows no significant differences between the HD and PD groups. Similar levels of ability to work, fulfillment in work, and overall contentment with life suggest that both modalities may have comparable impacts on patients' daily functioning. However, W. Wu et al. (2004) found that HD patients had greater improvements in physical functioning and general health perception compared to PD patients [21].

There are additional concerns for well-being and better QoL among dialysis patients based on different dialysis types. The absence of statistically significant differences in feelings of peace, having a reason for living, leading a productive life, and finding meaning and purpose indicates the universality of certain emotional and existential concerns among dialysis patients. The overall score for additional concerns, with no significant difference, reinforces the comparable impact of HD and PD on these dimensions.

Notably, the association between different employment statuses and the type of dialysis does not reach significance. The distribution of unemployed participants, students, housewives, professional employees, private employees, and retired individuals shows no statistically significant difference between the two dialysis groups. This suggests that both modalities may impact employment status similarly, highlighting the complex relationship between dialysis and employment.

Our study also presents the predictors of better QoL among both dialysis patient groups. For HD, important predictors include a high monthly income and being a smoker. The positive association with a high income aligns with previous research emphasizing the socioeconomic determinants of QoL. Similarly, Anees et al. (2018) found that socioeconomic factors, including education, employment, income, and funding, are important parameters affecting the QoL of kidney patients undergoing HD [22]. For PD, being male and not being a smoker show some trends, with age also playing a role. These findings underline the complex interplay of demographic and lifestyle factors in influencing QoL among dialysis patients. Similarly, Ibrahimou et al. (2019) [23] and Al Wakeel et al. (2012) [24] found that gender, age, nationality, living status, satisfaction with treatment, type of dialysis, and causes of ESRD are independent predictors of QoL.

Limitations

Our study has several limitations, including potential bias due to its cross-sectional design, reliance on self-reported data, and lack of long-term follow-up. Additionally, generalizability may be restricted as the sample is specific to certain regions and healthcare settings. We acknowledge that our study population may have limitations that affect broader applicability. While our findings are based on a specific cohort, we suggest further research to explore the applicability of our findings in diverse populations. This also includes investigating confounding variables or factors influencing patient outcomes, such as dialysis adequacy through objective measures.

## Conclusions

Our study contributes significantly to the understanding of QoL among dialysis patients in Saudi Arabia. By providing a comprehensive analysis of various dimensions of well-being and comparing these findings with previous studies, the research offers valuable insights into the complex interplay of demographic, treatment-related, and psychosocial factors. The nuanced differences observed between HD and PD highlight the need for personalized approaches in managing patients with ESRD. These findings contribute to ongoing efforts to enhance the quality of care and improve the overall well-being of dialysis patients.

## Appendices

Below is a list of statements that other people with your illness have said are important. Please circle or mark one number per line to indicate your response as it applies to the past 7 days.

**Table 7:** Functional Assessment of Chronic Illness Therapy – Spiritual Well-Being (FACIT-Sp), Version 4

Physical well-being		Not at all	A little bit	Some-what	Quite a bit	Very much
GP1	I have a lack of energy	0	1	2	3	4
GP2	I have nausea	0	1	2	3	4
GP3	Because of my physical condition, I have trouble meeting the needs of my family	0	1	2	3	4
GP4	I have pain	0	1	2	3	4
GP5	I am bothered by side effects of treatment	0	1	2	3	4
GP6	I feel ill	0	1	2	3	4
GP7	I am forced to spend time in bed	0	1	2	3	4
Social/family well-being						
GS1	I feel close to my friends	0	1	2	3	4
GS2	I get emotional support from my family	0	1	2	3	4
GS3	I get support from my friends	0	1	2	3	4
GS4	My family has accepted my illness	0	1	2	3	4
GS5	I am satisfied with family communication about my illness	0	1	2	3	4
GS6	I feel close to my partner (or the person who is my main support)	0	1	2	3	4
Q1	Regardless of your current level of sexual activity, please answer the following question. If you prefer not to answer it, please mark this box and go to the next section					
GS7	I am satisfied with my sex life	0	1	2	3	4
Emotional well-being						
GE1	I feel sad	0	1	2	3	4
GE2	I am satisfied with how I am coping with my illness	0	1	2	3	4
GE3	I am losing hope in the fight against my illness	0	1	2	3	4
GE4	I feel nervous	0	1	2	3	4
GE5	I worry about dying	0	1	2	3	4
GE6	I worry that my condition will get worse	0	1	2	3	4
Functional well-being						
GF1	I am able to work (include work at home)	0	1	2	3	4
GF2	My work (include work at home) is fulfilling	0	1	2	3	4
GF3	I am able to enjoy life	0	1	2	3	4
GF4	I have accepted my illness	0	1	2	3	4
GF5	I am sleeping well	0	1	2	3	4
GF6	I am enjoying the things I usually do for fun	0	1	2	3	4
GS7	I am content with the quality of my life right now	0	1	2	3	4
Additional concerns						
Sp1	I feel peaceful	0	1	2	3	4
Sp2	I have a reason for living	0	1	2	3	4
Sp3	My life has been productive	0	1	2	3	4
Sp4	I have trouble feeling peace of mind	0	1	2	3	4
Sp5	I feel a sense of purpose in my life	0	1	2	3	4
Sp6	I am able to reach down deep into myself for comfort	0	1	2	3	4
Sp7	I feel a sense of harmony within myself	0	1	2	3	4
Sp8	My life lacks meaning and purpose	0	1	2	3	4
Sp9	I find comfort in my faith or spiritual beliefs	0	1	2	3	4
Sp10	I find strength in my faith or spiritual beliefs	0	1	2	3	4
Sp11	My illness has strengthened my faith or spiritual beliefs	0	1	2	3	4
Sp12	I know that whatever happens with my illness, things will be okay	0	1	2	3	4
